# Efficacy of lung cancer screening at the American University of Beirut Medical Center

**DOI:** 10.3389/fonc.2023.1164574

**Published:** 2023-08-04

**Authors:** Tarek Harb, Anas Alhafi, Arafat H. Tfayli

**Affiliations:** Faculty of Medicine, American University of Beirut, Beirut, Lebanon

**Keywords:** lung neoplasms, early detection of cancer, secondary prevention, smoking, thoracic oncology

## Abstract

**Introduction:**

In Lebanon, a dedicated screening program for lung cancer is absent. Screening is largely based on the recommendation of an informed physician or the initiative of a patient. To better understand the situation, it is important to look at the available data on patients currently being screened for lung cancer in this country. Our aim in this study is to review the data and compare it with that in the literature as well as to assess the efficacy of the screening process followed.

**Methods:**

Our study accessed the electronic medical records of patients at the American University of Beirut Medical Center (AUBMC), a tertiary care center in Lebanon. We collected information on patients who underwent screening low-dose computed tomography (LDCT) scan between June 2019 and June 2021 inclusive. Records of all patients who underwent a non-contrast computed tomography (CT) scan at AUBMC during this period were collected and analyzed.

**Results:**

On average, our population had a 52.6 pack-year smoking history. Moreover, 47% of our population had an accurate pack-year reported, while 12% did not have enough information to even estimate their pack-year history. When looking at the accurate and estimated data, 5% of our population did not even meet the ≥20 pack-year smoking history. Eight patients had positive findings on the screening LDCT, which we defined as suspicious findings that require further workup (e.g., PET/CT or biopsy) or other significant incidental findings.

**Conclusion:**

A well-organized program for lung cancer screening in Lebanon is absent. Screening largely depends on the initiative of the physician or the patient. We were able to uncover multiple flaws in the screening method used, including poor documentation and follow-up. Although the screening method adopted retained some benefits in terms of detecting early malignancy, it lacked proper organization and was not ideal. A better, systematized screening program is needed to have optimal outcomes.

## 1 Introduction

Lung cancer is the leading cause of malignancy-related death in the United States and worldwide (1, 2). It has been long associated with a poor prognosis, with a 5-year survival rate of 21.7% (3). This may largely be attributed to patients being diagnosed at an advanced stage, with only 18% of cases diagnosed with a localized disease (3). Tobacco use has been established as the most important risk factor for lung cancer, as it accounts for nearly 90% of all lung cancer cases (3, 4).

Attempts at screening for lung cancer began in the early 1980s as evidenced by the Mayo Lung Project where they studied the use of chest X-rays (CXRs) to screen high-risk individuals every 4 months (5). Three large-scale studies which followed by combining CXRs and sputum cytology failed to show an improvement in overall survival, and it was concluded that better screening methods were necessary (6).

The National Lung Screening Trial (NLST) has shown that screening with low-dose computed tomography (LDCT) of the chest reduced lung cancer mortality by around 20% (7). Based on the results of this landmark study, as well as the Dutch–Belgian lung cancer screening trial (NELSON), the United States Preventive Services Task Force (USPSTF) established guidelines for lung cancer screening (8). The USPSTF currently recommends screening adults aged 50 to 80 years who have a 20-pack-year smoking history and currently smoke or have quit within the past 15 years with LDCT every year (grade B recommendation) (8). This recommendation replaced the 2013 USPSTF recommendation on screening for lung cancer which targeted an older age group (55 to 80 years) with more pack-years (30 pack-years). It was reasoned that screening persons at an earlier age and with fewer pack-years of smoking may help ameliorate racial disparities (8).

Lebanon has one of the highest prevalence of smoking in the Arab world, and the incidence of lung cancer in the country has increased from 25.3 to 35.6 cases per 100,000 from 2005 to 2015 (9). Between 2005 and 2016, lung cancer ranked second in male and female patients after prostate cancer and breast cancer, respectively, with a total of 11,570 incident cases and an average of 964 new cases per year. These rates are higher than those seen in neighboring Arab countries (10). Lung cancer is the leading cause of cancer-related mortality in the country (11). In the absence of dedicated screening programs in Lebanon, lung cancer screening is mainly based on the recommendation of a well-informed physician or the initiative of a well-informed patient (6).

To better understand the situation related to lung cancer screening in Lebanon, it is important to look at the data available on patients currently being screened for lung cancer in this country. Our aim in this study is to review the data and compare it with that in the medical literature as well as to assess the efficacy of the screening process followed.

## 2 Materials and methods

Our study accessed the electronic medical records of patients at the American University of Beirut Medical Center (AUBMC), a tertiary care center in Lebanon. No informed consent was needed.

To gather information on patients who underwent screening LDCT scans, records of all patients who underwent a non-contrast computed tomography (CT) scan at AUBMC between June 2019 and June 2021 inclusive were collected. We included patients aged >50 years whose imaging report or medical note indicated that the scan was ordered for screening purposes. Smoking history data were gathered *via* chart review either from the Epic “snapshot” or from the medical note of the ordering provider. We defined positive findings as suspicious findings that require further workup (e.g., PET/CT or biopsy) or other significant incidental findings. Data were analyzed on SPSS version 27. This study was approved by the Institutional Board Review (IRB) at AUBMC.

## 3 Results

A total of 10,988 records of patients who had a non-contrast CT scan of the chest done at AUBMC during our study period was collected. Of these, 476 records met the criteria for age (>50) and clinical indication (medical note or CT scan report clearly mentioning the screening purpose of the scan). After duplicates were removed, we were left with 419 records.

Of these 419 records, only 196 had an accurate pack-year reported. We were able to estimate the pack-year smoking history for 172 patients by conservatively assuming that patients started smoking by the age of 20 years (6, 12) and using the available reported information in the medical chart (e.g., one pack per day but no duration reported). In total, 51 patients did not have enough information on the number of pack-years reported.

**Table 1** presents a summary on the patients’ characteristics. The mean age for our patient population was 63.9 years. On average, our patient population had a 52.6-pack-year smoking history. Among them, 45% were male, 51% had hypertension, 30% had diabetes mellitus, 70% had dyslipidemia, and 13% had a history of cancer.

**Table 1:** Patients’ characteristics and findings.

*N* = 419	Result
Age in years, mean ( ± SD)	63.9 (7.1)
Cigarette pack-year best estimate	52.6 (38)
Male (%)	45
Hypertension (%)	51
Diabetes mellitus (%)	30
Dyslipidemia (%)	70
Personal history of cancer (%)	13
Positive findings warranting workup^a^ (%)	2
<20 pack-year smoking history (%)	5

a We defined positive findings as suspicious findings that require further workup (e.g., PET/CT or biopsy) or other significant incidental findings (e.g., other cancers). n = 368 for cigarette pack-year since 51 patients did not have adequately documented smoking history.

Eight patients had positive findings on imaging, which we defined as suspicious findings that require further workup (e.g., PET/CT or biopsy) or other significant incidental findings. **Table 2** shows the details of the stories of these cases and their management, in addition to two cases with notable screening mishaps (case numbers 9 and 10). Two of these eight patients did not follow their physicians’ recommendations appropriately. One patient had a lung malignancy resected. One patient was incidentally found to have a renal mass which turned out to be malignant, while another patient had an incidental thymoma resected. We also noted a few screening mishaps: One patient had two screening LDCTs in a span of 1 month as he sought the care of two different physicians. Another patient underwent a screening LDCT despite having a CT scan of the chest done for another reason 5 months ago.

**Table 2:** Summary of cases with positive findings.

Case number	Imaging results	Outcome
**1**	GGO consistent with either pneumonia or neoplastic Repeat imaging unchanged after a course of antibiotics	PET/CT was done and showed no FDG uptake. The patient failed to follow up despite a recommendation to repeat imaging in 6 months
**2**	A small exophytic lesion was incidentally noted at the lower pole of the right kidney. A 3-mm GGO was noted in the lung	The kidney lesion was resected as it was malignant. The patient followed with both his PCP and pulmonologist for his lung nodule, causing him to undergo two CT scans of the chest in the span of 1 month A follow-up CT scan at 9 months later showed stable findings
**3**	Spiculated pulmonary nodules in the right upper and lower lobes that were highly suggestive of malignancy	The patient refused further workup of his suspicious pulmonary nodules and was then lost to follow-up
**4**	8-mm spiculated GGO	The patient was adherent to her physician’s recommendation regarding screening and follow-ups. However, there was a 2-year gap between her screening LDCTs
**5**	Lung opacity suggestive of a malignant process PET/CT scan showed FDG-avid right upper lung nodule suspicious for a primary malignancy	Tissue sampling was recommended. The patient had poor lung function and thus underwent SBRT
**6**	Few small non-specific lung nodules Repeat imaging at 1 year later showed an increase in size of the sub-solid nodules	PET/CT or tissue sampling was recommended. The patient was late to follow up with physician (presented at 18 months later) and was asked to repeat imaging, which showed stable findings
**7**	Irregular nodular pulmonary opacity in the medial aspect of the left lower lobe Repeat imaging showed increased size	PET/CT scan which showed an increase in size; tissue sampling showed atypical cells suspicious for adenocarcinoma. Lobectomy was done and the pathology confirmed a diagnosis with a T1bN1M0 stage. The patient is alive and doing well
**8**	Few lung nodules An incidental lobulated soft tissue density in the anterior mediastinum was noted and can represent either thymic tissue or enlarged anterior mediastinal lymph nodes	PET/CT scan and eventual resection was done. Pathology showed type B2 thymoma
**9**	A 5-mm nodule was noted. At 15 months later, CT was done to rule out pneumonia and the result was normal At 5 months later, the patient had another LDCT, which showed stable findings	Unnecessary radiation exposure
**10**	Few nonspecific lung nodules	Screened although the patient is a passive smoker

FDG, (18)F-fluorodeoxyglucose; GGO, ground-glass opacity; LDCT, low-dose computed tomography; PCP, primary care physician; SBRT, stereotactic body radiation therapy.

## 4 Discussion

In any effective screening program, a fully functional system of scheduling and follow-up is needed. To illustrate, the system followed by the Centers for Medicare and Medicaid Services (CMS) can serve as an example (13): the CMS uses the same eligibility criteria as the USPSTF except for age (55–77 years instead of 50–80 years). To enroll in the lung cancer screening service, the person should receive counseling in a shared decision-making visit. The counseling should include discussions regarding the benefits and harms of screening, follow-up diagnostic testing, over-diagnosis, false positive rate, and total radiation exposure. The importance of adherence to annual lung cancer LDCT screening should be emphasized. The impact of comorbidities and ability or willingness to undergo diagnosis and treatment should also be discussed. Counseling on the importance of maintaining cigarette smoking abstinence (if formerly a smoker) or the importance of smoking cessation (if currently a smoker) is mandatory in this visit as well. A close follow-up system is in place to ensure adherence to yearly screening LDCTs and continuous counseling about smoking cessation, making all interventions required to stop or prevent smoking initiation readily available (13). Besides that, the medical records of the people enrolled in the service should be accurate and updated. The accurate number of pack-years and smoking status (current or former and since how many years) should be easily accessible and stated in the LDCT order. Finally, it is required to have a standardized lung nodule identification, classification, and reporting system.

In Lebanon, a dedicated lung cancer screening program is not available. Screening is largely based on the recommendation of a well-informed physician or the initiative of a well-informed patient (6). This method of screening has many drawbacks, some of which were revealed in our study. First, we noticed poor documentation of smoking history. In fact, only 47% of our patient population had an accurate pack-year reported, while 12% did not have enough information to even estimate their pack-year history. When looking at the accurate and estimated data, 5% of our population did not even meet the ≥20 pack-year smoking history (12, 14), in addition to one patient who was a nonsmoker but was reported as a heavy passive smoker.

It is important to note the characteristics of our patient population. Only 45% of our population were male patients, which is interesting because sociodemographic studies revealed a prevalence of smoking among male individuals to be twice as much as in female individuals in Lebanon (15, 16). This might reflect that female individuals are more compliant with their cancer screening recommendations than male individuals (17, 18). At an average age of 63.9 years, the prevalence of hypertension and diabetes mellitus in our population is similar to other studies done in Lebanon, while the prevalence of dyslipidemia was almost twice as high (19–21).

Our definition of positive findings was set due to the lack of uniform reporting of nodule sizes and characteristics in the medical records and imaging reports retrieved. In total, 2% of our patients met this definition. Our result is similar to that present in the NELSON trial which yielded a 2.6% positive rate, although they used a complex categorization system based on nodule size, characteristics, and growth rate (22). The NELSON trial also details the management protocol and follow-up schedule for each category.

Our result, however, was quite different than the rate yielded in the NLST which reported that 24% of their patients had positive findings. This discrepancy is likely attributed to the difference in the definition of positive findings. In fact, the NLST included any non-calcified nodule greater than 4 mm in size, any lung consolidation or obstructive atelectasis, nodule enlargement, or nodules with suspicious changes in attenuation in their definition (23). Data regarding rates of screening positive findings in similar populations is currently not available (6).

In summary, screening for lung cancer without a dedicated screening program retained some of its benefits by detecting early malignancy, but for the large part, it was disorganized and untidy.

To have optimal outcomes of lung cancer screening, it is important to have a proper formed system like the CMS system illustrated above to ensure accurate records of patients who meet the criteria and close follow-ups for those in need of monitoring, other diagnostic tests, or treatment. Implementing such a program ensures proper assessment for lung cancer screening eligibility.

Our study mainly relied on data collection from electronic health records and was based on a 2-year window which might have restricted us from capturing the entire picture of the screening process. Poor documentation in the patients’ medical records might also have affected our results. Since there was not a standardized reporting system to identify nodules, we were unable to adequately summarize the nodule findings. Notably, the timeframe chosen to collect the medical records coincided with the COVID-19 pandemic, and this might have affected our observations.

In Lebanon, a well-organized screening program for lung cancer screening is absent. Screening largely depends on the initiative of the physician or the patient. Our study looked at the lung cancer screening data available at AUBMC, a tertiary care center. We were able to uncover multiple flaws in the screening method used, which included poor documentation and follow-up. Although the screening method adopted retained some benefits in terms of detecting early malignancy, it lacked proper organization and was not ideal. A better, systematized screening program is needed to have optimal outcomes.

## Data availability statement

The raw data supporting the conclusions of this article will be made available by the authors without undue reservation.

## Ethics statement

The studies involving human participants were reviewed and approved by the Institutional Review Board of the American University of Beirut. Written informed consent for participation was not required for this study in accordance with the national legislation and the institutional requirements.

## Author contributions

TH contributed to the data collection, data analysis, literature review, and manuscript writing. AA contributed to the data collection, data analysis, literature review, and manuscript writing. AT supervised the data collection and analysis and revised, commented on, and approved the final manuscript. All authors contributed to the article and approved the submitted version.
